# Editorial: Neurodegeneration and cognitive impairment after traumatic brain injury

**DOI:** 10.3389/fnins.2025.1552527

**Published:** 2025-01-29

**Authors:** Tao Liu, Li Ma, Yu Chen, Rongcai Jiang

**Affiliations:** ^1^Department of Neurosurgery, Xuanwu Hospital, Capital Medical University, Beijing, China; ^2^Department of Neurosurgery, Tianjin Medical University General Hospital, Tianjin, China; ^3^The George Institute for Global Health, Faculty of Medicine, University of New South Wales, Sydney, NSW, Australia; ^4^Department of Neurosurgery, University of Pittsburgh Medical Center, Pittsburgh, PA, United States; ^5^Department of Neurosurgery, Beijing Tiantan Hospital, Capital Medical University, Beijing, China

**Keywords:** traumatic brain injury, cognitive impairment, neurodegeneration, subdural hematoma (SDH), nervous system

Traumatic brain injury (TBI) remains a critical public health concern. Despite advancements in acute care and trauma management that have improved survival rates, the long-term effects, including neurodegeneration and cognitive decline, pose substantial challenges (Liu et al.). TBI initiates a cascade of pathological processes that extend beyond the primary injury, resulting in progressive and enduring damage. Secondary injury, such as inflammation, oxidative stress, and excitotoxicity, are central drivers of neurodegeneration and may lead to chronic traumatic encephalopathy (CTE), Alzheimer's disease, and other neurodegenerative disorders. Growing evidence also underscores the cumulative damage caused by repeated mild TBI (mTBI), highlighting the urgent need for greater awareness and targeted research to address its long-term consequences (Liu et al.).

The causes of deficits following TBI are complex and multifactorial, involving direct neuronal damage, disruption of brain functional networks, and systemic factors. The heterogeneity of TBI (spanning variations in injury mechanisms, severity, and individual differences) further complicates research and clinical management. This underscores the urgent need for standardized protocols in diagnosis, treatment, and outcome assessment. Additionally, the progressive nature of neurodegeneration necessitates long-term follow-up, which remains challenging in both clinical practice and research settings. Recent advances have significantly enhanced our understanding of TBI-related neurodegeneration and cognitive impairment. Key areas of progress include neuroinflammation, tau protein pathology, waste clearance mechanisms via the intracranial lymphatic system, and advanced neuroimaging techniques for detecting neurodegenerative changes. These findings have not only clarified underlying mechanisms but also identified promising therapeutic targets, paving the way for future research.

This Research Topic addresses these critical challenges by highlighting the latest developments in the diagnosis, treatment, and prevention of TBI-related neurodegeneration, while providing new insights into its underlying mechanisms.

Chen et al. identified significant differences in post-concussion syndrome (PCS) risk factors between younger and older patients. Chronic pain and headaches were more prevalent in younger individuals, while the APOE ε4 allele emerged as a major risk factor for older adults. Additionally, women and individuals with specific genetic variations demonstrated an elevated PCS risk. These findings highlight the need for age-specific approaches to better understand and manage TBI outcomes. Liu et al. reviewed the epidemiology, causes, risk factors, and management strategies for cognitive impairment among Chinese TBI patients. They proposed improvements from basic research to clinical practice, aiming to enhance prevention and treatment efforts. The authors also emphasized the importance of standardized, systematic investigations in future studies. Caldas et al. addressed a critical gap in the literature with a prospective, longitudinal observational study examining the link between acute cerebrovascular dysfunction and cognitive outcomes 1-year post-TBI. Their work underscores the need for long-term follow-up and comprehensive cerebrovascular function assessments. Promising progress has also been reported in multicenter clinical trials investigating therapies to improve cognitive outcomes following TBI. These include edaravone dexborneol, oxiracetam, piracetam, and compound porcine cerebroside injection, offering encouraging advancements for patient care (Fu et al., 2024; Malík and Tlustoš, 2022; Liu et al., 2024b; Guangliang Bian, 2020; Malykh and Sadaie, 2010; Liu et al., 2024a).

The brain's lymphatic drainage system, comprising the glymphatic system and meningeal lymphatic vessels (MLVs), plays a vital role in clearing waste after TBI. Recent research has focused on targeting this system to enhance cognitive recovery and overall outcomes (Liu et al., 2023; Jiang et al., 2024). Zhuo et al. suggested that glymphatic imaging markers, such as the enlarged perivascular space (ePVS) burden and the ALPS index, may provide valuable insights into TBI recovery and guide targeted interventions. Similarly, Qin et al. proposed that low-frequency amplitude (ALFF) analysis using resting-state functional MRI (rs-fMRI) could help predict treatment outcomes in patients with disorders of consciousness. These studies reinforce the potential of advanced neuroimaging and biomarkers for early diagnosis, monitoring, and precise management of TBI-related neurodegeneration.

Chronic subdural hematoma (cSDH) often occurs after mild brain injury, which may lead to cognitive impairment. cSDH has long been a subject of research interest, particularly following two recent randomized controlled trials (RCTs) in *The New England Journal of Medicine* exploring middle meningeal artery embolization as a treatment option (Davies et al., 2024; Liu J. et al., 2024). These studies sparked debate over whether surgery is necessary for patients with mild or non-progressive symptoms. For decades, surgery has been the mainstay treatment for cSDH, offering symptom relief but carrying risks, including mortality and a 10% recurrence rate (Liu et al., 2024d). cSDH primarily affects older adults, and in low- and middle-income countries, the financial burden of surgery remains a major obstacle. This has fueled global efforts to identify effective non-surgical alternatives, particularly for patients unable to undergo surgery. A 2018 RCT published in *JAMA Neurology* highlighted atorvastatin as a promising non-surgical therapy for cSDH, sparking significant interest (Jiang et al., 2018). Statin therapy is now widely used in China, and ongoing real-world studies are expected to provide further evidence, offering hope for improved treatment strategies (Liu et al., 2024c).

The discussions surrounding cSDH reflect broader challenges in TBI management, a field that remains riddled with unresolved questions. However, optimism persists as ongoing research seeks to optimize care. In the acute phase, balancing intracranial pressure monitoring and surgical interventions to reduce mortality while preventing long-term neurological impairments is a key clinical focus. Similarly, during rehabilitation, pharmacological treatments and cognitive training must be carefully tailored to avoid causing further harm to the nervous system. Adhering to the principle of “Primum Non Nocere,” also called first, do no harm; while prioritizing individualized care and scientific rigor is essential for improving long-term outcomes for TBI patients.

In summary, the relationship between TBI and neurodegenerative diseases remains a critical area of research, with significant knowledge gaps surrounding mechanisms, diagnostics, treatments, and individual variability. The roles of genetic, biological, and environmental factors in TBI-related neurodegeneration are still poorly understood. Additionally, the sequence, rate, and extent of neuropathological progression or resolution remain speculative due to the lack of sensitive and specific biomarkers. Exploring the interplay between TBI complications and neurodegeneration is crucial for advancing the field. Addressing the complexities of TBI requires interdisciplinary collaboration and global research initiatives. Continued innovation and collective efforts are essential to uncover effective solutions, bringing us closer to addressing this pressing public health challenge and improving outcomes for affected individuals worldwide.
